# Our Position

**Published:** 1889-12

**Authors:** 


### Editorial.

### OUR POSITION.

The *Items*, in a short editorial on "its position," in which it defines the purposes of "its" company publisher to be "to bring to the average dentist the greatest amount of practical information possible, whether original or selected, and of raising his standard morally, socially, and professionally," says further:

"Journals presenting more scientific and exhaustive articles are also doing a good work, and it is difficult to see how a progressive dentist can do without one. Not to disparage any of our dental periodicals—for American dentists should be proud of all of them—we are pleased to make special mention of the high standing and progressive character of the International Dental Journal, edited by Dr. W. X. Sudduth, of Philadelphia. This magazine was admirably conducted while under the supervision of Dr. Barrett; but it has lost none of its vim and thoroughness and dignity under the management of its present editor. Every number shows he is putting into it his very life."

We feel like thanking the genial editor of the *Items* for these kindly comments upon the course of the Journal. It is our aim to try and inculcate in the minds of our readers a higher professional tone in business as well as professional ethics, and lead up to an understanding of the principles which underlie *dental science* which are identical with those relating to medicine. When these are fully understood, and the "why" of things rather than the "how" is the query upon the lips of speakers in our dental conventions, then will dental science merge into medical science, and dentistry be a specialty in medicine, which is her rightful heritage. Surgery occupied a position similar to that from which dentistry is now rapidly emerging. It was by looking to medicine for instruction in the principles that underlay surgical practice that surgery became adopted as a specialty in medicine, and so will dentistry. Our field is to deal with these principles which, when they are fully understood, will indicate the method of procedure in every instance. He is the best dentist who can explain to himself the reason why he adopts a certain line of treatment, and has the mechanical ability to carry out the suggestions derived from the study of the cause of the pathological conditions existing in any given case. We are free to admit, however, that a very fair dentist may be made by teaching a man the "how" of dentistry; but the same individual would have made a much better dentist had the "why" at the same time been included in his instruction. If you feel that your education has been neglected in the principles of dentistry, do not conclude that it is too late to learn, but attend at once to it that you are put in possession of the means of self-instruction which is to be found in our dental literature, which may be taken to your fireside and perused at leisure. There is not a dental journal published in this country but what contains suggestions which, if adopted, will many times repay the price of the subscription, and of no other journal can this be more truthfully said than of the INTERNATIONAL DENTAL JOURNAL, which is replete with *original* matter, fresh from the offices and laboratories of the best men in the profession.

The present number closes volume ten, the first complete one under our management. We look back with conscious pride at the steady improvement made during the past year. Each number has been an advance upon its predecessor, and it shall be our aim to continue to add to the value of its contents and the high standard of its mechanical execution; but in order to do this we must have the hearty support of the profession for whom the JOURNAL is published.

We are sorry to say that some are in arrears for this volume, and unless we hear from them between now and the issuing of the January number, we shall be compelled to drop them from our list, a consummation we should most heartily deprecate. We therefore make the following offer: to all those who know themselves to be behind in their subscription, and who will send us a check for five dollars for two years' subscription, we will forward them by mail, postage paid, our new *International Binder*, for volume ten, free. It consists of a cloth case for binding the complete volume, and printed in gold on the back are the words, "THE INTERNATIONAL DENTAL JOURNAL, *Volume X.*," and the date "1889;" also, "Published by the International Dental Publication Company, Philadelphia." On the side are the words, "The International Dental Journal, edited by W. Xavier Sudduth," also in gilt. These cases can be sent to any ordinary bookbinder, and, for a trifling outlay, the separate numbers will be sewed and pasted in the already prepared backs. These cases will be provided annually in order to secure uniformity in binding for succeeding numbers.

